# Natalizumab treatment of multiple sclerosis in Spain: results of an extensive observational study

**DOI:** 10.1007/s00415-012-6414-9

**Published:** 2012-01-31

**Authors:** O. Fernández, C. Oreja-Guevara, R. Arroyo, G. Izquierdo, J. L. Pérez, X. Montalban

**Affiliations:** 1 Multiple Sclerosis Unit, La Paz University Hospital, Madrid, Spain; 2 Multiple Sclerosis Unit, San Carlos Clinic Hospital, Madrid, Spain; 3 Neurology Department, Virgen Macarena University Hospital, Seville, Spain; 4 Neurology, Sierrallana Hospital, Torrelavega, Cantabria, Spain; 5 Neuroimmunology Unit, Vall d’Hebron University Hospital, Barcelona, Spain; 6 Institute of Clinical Neurosciences, Carlos Haya Regional University Hospital, Avenida Carlos Haya s/n, 29010 Málaga, Spain

**Keywords:** Multiple sclerosis, Natalizumab, Observational study, Spain, Relapse, Disability progression

## Abstract

Natalizumab has been shown to be effective in pivotal clinical trials in multiple sclerosis; however, the patients in whom treatment is indicated in clinical practice have a different clinical profile from those included in the clinical trials. The aim of this study is therefore to collect data on natalizumab use in everyday clinical practice in Spain. The 86 participating centers throughout Spain submitted data on disease characteristics at baseline and after treatment. Valid data were available for 1,364 patients (69.3% women, 86.9% with relapsing–remitting disease). Ninety-three percent had received prior therapy for multiple sclerosis. For the 825 patients on treatment for at least a year, the annualized relapse rate (ARR) decreased from median 2.0 [mean 2.01, 95% confidence interval (CI) 1.92–2.11] in the year prior to natalizumab to 0.0 (mean 0.25, 95% CI 0.21–0.29) at 1 year (*p* < 0.001). The Expanded Disability Status Scale (EDSS) score decreased from median 3.5 at baseline (mean 3.71, 95% CI 3.60–3.82) to 3.0 (mean 3.37, 95% CI 3.25–3.49) (*p* < 0.0001). The discontinuation rate was 14%. One patient discontinued natalizumab due to progressive multifocal leukoencephalopathy (PML) and another due to probable PML (subsequently confirmed). Although our patients had more severe disease than those in the pivotal study, a similar reduction in ARR was observed. This finding is in line with previous observational studies. The effect was independent of baseline EDSS.

## Introduction

Natalizumab is a monoclonal antibody that interferes with leukocyte trafficking into the central nervous system by antagonizing the α_4_ subunit of integrin expressed on the surface of activated T-cells [1, 2]. Such an action is thought to reduce the inflammatory component of multiple sclerosis (MS), particularly during the early relapsing–remitting phases of the disease [3]. A number of randomized controlled phase II and phase III studies have shown natalizumab to be effective at reducing the annualized relapse rate (ARR) and also disease progression as measured using the Expanded Disability Status Scale (EDSS). In most cases, a substantial reduction in disease activity according to magnetic resonance imaging (MRI) measures has also been documented [4–7]. Natalizumab has been on the market in Spain since 2007, and as of July 2010, 1,865 patients had been treated.

Natalizumab was approved largely on the strength of the pivotal AFFIRM study, which compared natalizumab infusions with placebo infusions in patients who for the most part had not received prior MS therapy [6]. However, in view of the risk of progressive multifocal leukoencephalopathy (PML)—developed by two patients in the SENTINEL study [7], which compared natalizumab versus the combination of natalizumab plus interferon β-1a IM—the indication was limited to patients who have failed front-line therapy or who have an aggressive form of the disease. As a consequence, patients treated in clinical practice tend to have more severe disease than those enrolled in the AFFIRM study [8–12].

The aim of this multicenter, retrospective study is to collect data on patients treated with natalizumab in Spain in everyday clinical practice and to compare the patient and disease characteristics both with those published in clinical trials and with other observational studies.

## Patients and methods

All Spanish centers that had treated at least one patient with natalizumab before 31 May 2010 were invited to participate in the study. To be included, patients had to be diagnosed with MS and have received at least one dose of natalizumab prior to this cut-off date. All patients gave written informed consent for their data to be used in the study, which was approved by the Ethics Committees of the participating centers and notified to the Spanish Drug Agency. The investigators retrospectively filled out electronic data-collection forms (an Excel spreadsheet) for natalizumab-treated patients from their clinical records. Data were transferred anonymously from the returned Excel forms to the database to safeguard the privacy of the patients.

The data-collection forms included sections for demographic data (sex and date of birth), baseline disease data [year of diagnosis of MS, prior treatments, clinical form of MS prior to initiation of natalizumab treatment, EDSS score prior to treatment with natalizumab, ARR prior to treatment with natalizumab, and MRI data (presence of T1 Gd-enhancing lesions and T2-hyperintense lesions prior to treatment)], details of natalizumab treatment (number of natalizumab perfusions), and disease data after initiating treatment (EDSS score at 6 and 12 months after starting treatment, ARR after 12 months of treatment, and MRI data after 12 months of treatment). In addition, safety data were collected (adverse drug reactions, neutralizing antibodies, hypersensitivity reactions, and reason for treatment withdrawal when applicable). Data from the forms were collected and analyzed by a contract research organization (Phidea Marvin, Madrid, Spain).

### Statistical analysis

Descriptive statistics were calculated for patient data [mean, standard deviation (SD), minimum, median, maximum for continuous variables, and percentages for categorical variables]. Variables were compared using the McNemar test or the Wilcoxon test as appropriate, and statistical significance was set at *p* < 0.05. Analysis was performed with the SAS program.

## Results

### Data capture

Data were collected from 86 Spanish centers, corresponding to an estimated 56% of all Spanish centers where natalizumab is prescribed and accounting for approximately 85% of natalizumab prescriptions in Spain.

Data were available for 1,415 patients. Fifty-one patients were excluded from the subsequent analyses because of inconsistency in the data (36 patients), first administration of natalizumab before the patient’s 18th birthday (14 patients), or missing number of infusions (one patient). The final number of patients analyzed was therefore 1,364.

### Patient characteristics

In the year prior to treatment the median ARR was 2.0 (range 1–14) (mean 2.01, 95% CI 1.94–2.08). The median EDSS score at baseline was 3.5 (mean 3.77, 95% CI 3.68–3.86). Most patients had EDSS between 2 and 3 [478/1,318 (36.3%)] and between 3.5 and 4.5 [349/1,318 (26.5%)]. Fifty-three patients (4.0%) had baseline EDSS >6 points. The patients included predominantly had the relapsing–remitting form (87%); the remainder had secondary progressive MS with relapses.

A large majority of patients had received prior MS treatment [1,268/1,363 (93.0%)], and the median duration of this prior treatment was 52 months (range 0–258 months). Subcutaneous (SC) interferon beta 1a was the most common prior treatment, followed by interferon beta 1b SC (Table 1). In total, 603 patients (44.2%) had received only one prior treatment while 665 (48.8%) had received two or more prior treatments (up to a maximum of six). Overall, 113 (8.9%) had received prior immunosuppressive therapy (mainly mitoxantrone). The reasons for switching to natalizumab were cited as lack of efficacy in 75.2%, poor tolerability in 18.5%, and patient decision in 6.2% (more than one reason possible). The median number of natalizumab infusions received was 16.0 (range 1–50). In total, 287 (21.0%) had received natalizumab for more than 2 years. One patient, who had been enrolled in a clinical trial with natalizumab, had received natalizumab for more than 4 years (natalizumab has only been available in Spain since 2007).

**Table 1:** Demographic and baseline disease characteristics

Variable	*n* = 1,364
Sex	
Female	944 (69.3%)
Male	418 (30.7%)
Mean ± SD age on diagnosis of MS, years	29.6 ± 8.50
Mean ± SD time since diagnosis of MS, years	9.62 ± 5.65
Mean ± SD age on starting natalizumab, years	39.2 ± 8.95
Clinical form of MS prior to starting natalizumab	
Relapsing–remitting	1,173 (86.8%)
Secondary progressive	178 (13.2%)
Prior MS treatments	
No	95 (7.0%)
Yes	1,268 (93%)
One prior treatment	603 (44.2%)
Two prior treatments	431 (31.6%)
>2 prior treatments	234 (17.1%)
Interferon beta 1a SC	655 (51.7%)
Interferon beta 1b SC	447 (35.3%)
Interferon beta 1a IM	364 (28.7%)
Glatiramer acetate	330 (26.0%)
Immunosuppressants	113 (8.9%)
Mitoxantrone	98 (7.7%)
Methotrexate	7 (0.6%)
Cyclophosphamide	9 (0.7%)
Azathioprine	4 (0.3%)
Other treatments	120 (9.5%)
EDSS score in year prior to treatment	3.0 (0.0–8.0)
Annualized relapse rate in year prior to starting natalizumab	2.0 (1–14)
No. of T1 Gd-enhancing lesions prior to natalizumab	
0 lesions	530 (47.7%)
1–5 lesions	467 (42.0%)
6–9 lesions	49 (4.4%)
>9 lesions	66 (5.9%)

*EDSS* Expanded Disability Status Scale, *IM* intramuscular, *MS* multiple sclerosis, *SC* subcutaneous

### Safety data: discontinuation of treatment

Overall, 176/1,249 patients (14.1%) discontinued treatment. Reasons for discontinuation were cited as lack of efficacy [37/1,249 (3.0%)], tolerability [36/1,249 (2.9%)], and patient decision [36/1,249 (2.9%)]. Other reasons for discontinuation were pregnancy or desire to become pregnant [15/1,249 (1.2%)], neutralizing antibodies [34/1,082 (3.1%)], and hypersensitivity [46/1,284 (3.6%)] (more than one reason possible).

Most discontinuations occurred in the first year [103/176 (58.5%), of which 55 (31.3%) occurred within 6 months of starting natalizumab]. Discontinuations due to lack of efficacy tended to occur between 6 and 12 months after starting treatment [19/37 (51.4%)], whereas discontinuations due to tolerability tended to occur in the first 6 months [20/36 (55.6%)].

Of note was one discontinuation due to PML after 13 infusions and another due to suspected PML (later confirmed) after 14 infusions. The patient with confirmed PML had the secondary progressive form of the disease (EDSS of 5.5 on starting treatment) and had undergone bone marrow transplant prior to initiating treatment with natalizumab. The patient with suspected PML had the relapsing–remitting form, but his EDSS score had increased from 4.5 in the year prior to treatment to 5.5 on initiating treatment. She did not have a history of treatment with immunosuppressants.

Limited data are available on patients after discontinuation of natalizumab (*n* = 112). Of the 82 who were documented to have received treatment, 78 had details available. The most common treatments after discontinuation of natalizumab were glatiramer acetate [16/78 (20.5%)] and mitoxantrone [13/78 (16.7)]. Reactivation of the disease was reported in 47 out of 136 patients with data available (34.6%) during follow-up. Reactivation was in the form of relapses in 93%, MRI lesions in 31%, and increased EDSS in 50%, and almost half (47.6%) had more than one form of disease reactivation. The median time to disease reactivation was 3.5 months (range 1.0–24.0 months).

### Safety data: hypersensitivity reactions, neutralizing antibodies, and concomitant infections

Hypersensitivity reactions were reported in 46 patients (3.6%), although as noted above, only ten of these patients actually withdrew due to such reactions. Thirty-four patients, that is, 2.5% of the entire population and 3.1% of the 1,082 with information on neutralizing antibodies, had positive status in an antibody test. We note, however, that patients were only tested in the event of suspicion of neutralizing antibodies and that this test had only been performed in approximately 20% of these patients. Concomitant infections were reported by 144 patients out of 1,283 with data available (11.2%) during natalizumab treatment; none of these were considered severe.

### Efficacy outcomes

**Fig. 1:**
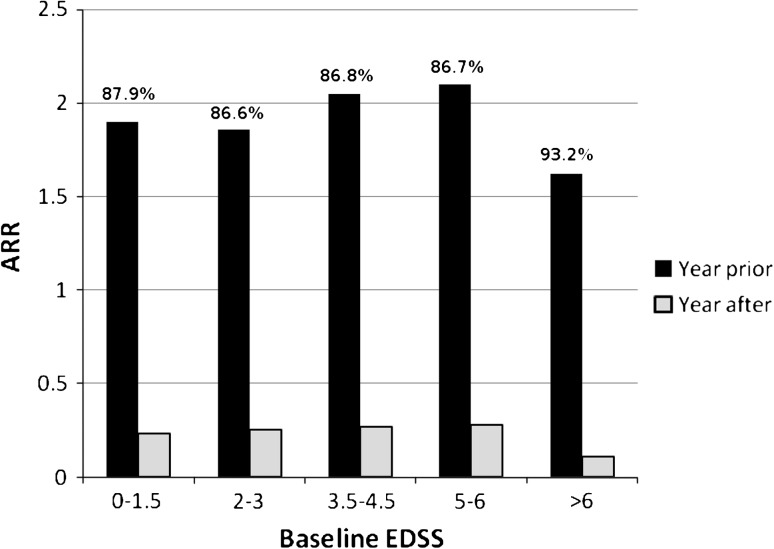
Change in annualized relapse rate (year prior to and year after starting natalizumab treatment for all patients in treatment for at least 12 months) by baseline Expanded Disability Status Scale score. Percentages indicate relative reductions in the ARR between the year before treatment and the year on treatment

For patients who completed at least 12 months of treatment, the median ARR decreased from 2.0 (mean 2.01, 95% CI 1.92–2.11) in the year prior to treatment to 0.0 (mean 0.25, 95% CI 0.21–0.29) during the 12-month treatment period [*p* < 0.0001 (McNemar test)] (Table 2). On stratification by baseline EDSS, similar decreases were observed across the different initial disease severities (Fig. 1). In addition, the percentage of patients with at least one relapse decreased from 89% in the year prior to treatment to 20% in the year after starting treatment.

**Table 2:** Efficacy outcomes for patients on treatment for at least 12 months

Outcome measure	Baseline		At 12 months/during 12-month treatment period	
	*n*	Value	*n*	Value
Annualized relapse rate^a^	826	Median 2.0 (0–14)	825	Median 0.0 (0–4)^b^
		Mean 2.01 (1.92–2.11)		Mean 0.25 (0.21–0.29)
Percentage of patients with at least one relapse^a^	826	733 (88.7%)	825	168 (20.4%)
Basal EDSS score	839	Median 3.50 (0–8.5)	839	3.00 (0–7.5)^c^
		Mean 3.71 (3.60–3.82)		Mean 3.37 (3.25–3.49)
Patients with Gd-enhancing lesions at baseline	563	289 (51.3%)	563	33 (5.9%)

Results presented as median (range), mean (95% confidence interval), or absolute number (%)

*EDSS* Expanded Disability Status Scale

^a^In the year prior to baseline

^b^ *p* < 0.001 versus baseline (Wilcoxon test)

^c^ *p* < 0.001 versus baseline (McNemar test)

The EDSS score also decreased [from median 3.5 at baseline to 3.0 at 12 months (*p* < 0.0001, Wilcoxon test), mean from 3.71 (95% CI 3.60–3.82) at baseline to 3.37 (95% CI 3.25–3.49) at 12 months] (Table 2). Twenty-four percent of the patients showed improvement in EDSS (defined as a decrease of ≥1.0 point) and only 6% showed worsening (defined as an increase of ≥1.0 point) between baseline and 1 year after treatment [Table 3, *p* < 0.0001 for the differences (McNemar test)], the rest (70%) remaining stable.

**Table 3:** Summary of change in disease status according to Expanded Disability Status Scale score

Change in disease status	Time period relative to baseline		
	−12–0 months	0–6 months	6–12 months
Improvement	3.55	17.52	23.77
Stability	68.14	78.68	69.98
Worsening	28.31	3.8	6.25

Improvement defined as decrease ≥1 point on Expanded Disability Status Scale, stability as <1 point change, and worsening as increase ≥1 point over time. Only patients in treatment for 12 months included. The differences were statistically significant [*p* < 0.0001 (McNemar test)]

Less than half the patients were free of Gd-enhancing lesions in the year prior to starting natalizumab treatment (Table 2), whereas in the year following start of treatment, 96.4% were free of such lesions [*p* < 0.0001 (McNemar test)]. Only 23/289 patients (8.0%) with baseline Gd-enhancing lesions still had Gd-enhancing lesions after 1 year of treatment, whereas 10/274 patients (3.7%) without baseline Gd-enhancing lesions developed such lesions after 1 year of treatment. After 1 year of treatment, 84.5% of patients were free of new T2-hyperintense lesions.

We defined a variable “disease free” as patients without any of the following: worsening EDSS (≥1 point increase), presence of relapses, and presence of Gd-enhancing and new T2-hyperintense lesions on MRI. According to this definition, 63% were free of disease during the year of treatment with natalizumab.

Of the 178 patients (13%) who had a secondary progressive (SP) form of the disease, 102 received treatment with natalizumab for at least 12 months. For these patients, the median ARR of 1.0 (mean 1.27, 95% CI 1.07–1.48) the year prior to the treatment decreased to 0 (mean 0.13, 95% CI 0.05–0.21) after 1 year of treatment. The median EDSS score at baseline for these patients was 6.0 (mean 5.54, 95% CI 5.33–5.74), and at 12 months showed little variation (median 6, mean 5.32, 95% CI 5.06–5.58).

Thirty-three percent of the SP patients showed an improvement in EDSS and only 15% showed worsening between baseline and 1 year after treatment; the rest (53%) remained stable. In total, 73% of the SP patients were free of disease (according to the above definition) during the year of treatment with natalizumab.

## Discussion

Although randomized clinical trials provide the highest level of evidence for the efficacy of a given drug, their restrictive inclusion criteria and necessarily rigid protocols may compromise their relevance for clinical practice. Moreover, certain safety signals may only become apparent when the drug has been administered to large numbers of patients and so may not be detected in clinical trials during drug development. It is therefore important to conduct observational studies with a view to providing data from real clinical practice. In the case of natalizumab, the need for “real-life” data is particularly pertinent given that the final approved indications limit use of the drug to patients who have failed front-line therapies and those with highly active disease. In the present study, we retrospectively collected data from a large sample of natalizumab-treated patients, accounting for approximately 85% of patients treated with this drug in Spain.

As expected, ARRs in the year prior to treatment and baseline EDSS were higher in our study than those reported in the pivotal AFFIRM trial [6]. Nevertheless, despite a higher pretreatment ARR, the ARR in our study in the year after treatment was similar to that seen in the AFFIRM trial. Of note is that the decrease in the number of relapses is observed regardless of baseline EDSS, even in patients with baseline EDSS >6.0 (the AFFIRM trial did not include any patients with baseline EDSS >6.0). Also of note was a significant decrease in EDSS for patients who completed 12 months of treatment (from median EDSS 3.5 to 3.0 and from mean EDSS 3.77 to 3.38).

A number of points need to be addressed when interpreting the results of an observational study; for example, patients who are included may be those who the treating physicians consider will do well on treatment. In addition, patients who do not complete 12 months of treatment with natalizumab may be more likely to have poor outcomes if they stay on therapy, and these patients are often not considered in the final efficacy analysis of observational studies. In our study, 37 patients withdrew due to lack of efficacy (among other possible reasons). Even if all patients who discontinued treatment (14% of total sample analyzed) were considered as efficacy failures, rough calculations based on a sample that either completed 12 months of treatment or discontinued treatment suggest that the treatment effect would still be considerable (approximately 70% free of relapses, which is closer to the 77% observed in the active treatment arm of the AFFIRM trial).

Another possible confounder in the interpretation of observational studies with no control arm is that the treatment effect may be partly explained by regression to the mean. Thus, patients are likely to start natalizumab treatment during moments of high disease activity and this activity would subside to a certain extent anyway, regardless of treatment. In addition, we should also bear in mind the tendency for fewer exacerbations as MS progresses, although the period of observation here was probably too short to have a major impact on the results. In our study, the decrease in ARR observed was 87.6% when calculated relative to the previous year for all patients and 75.6% when calculated for patients on treatment for 1 year. These values are very similar to the relative decrease in ARR at 1 year observed between the active treatment and placebo arm of the AFFIRM trial (68%), so that the effect of regression to the mean was probably small in our study. Particularly interesting are the results obtained in patients already in the SP phase of the disease.

The retrospective nature of data collection may also introduce a bias into the study results. Given these caveats in the interpretation of observational studies, it is reassuring that other observational studies performed in similar patient populations in Germany, Switzerland, Denmark, Italy, France, Sweden, and Spain show similar results [8–17]. Table 4 summarizes the baseline characteristics of the principal European observational studies. As in our study, patients appear to have more severe disease than those patients enrolled in the AFFIRM trial, reflecting the approved indications for natalizumab. Patients in the Danish study by Oturai et al. [9], however, appeared to have a higher ARR in the year prior to treatment and a higher baseline EDSS than the rest. This perhaps suggests that, in Danish clinical practice, natalizumab is reserved for patients with more active or severe disease. It is encouraging to see that, despite different methodologies and presumably some variation in clinical practice and treatment protocols in the different countries, good efficacy is observed in terms of reducing ARR and EDSS (Table 5). In all cases, the ARR is reduced to 0.2–0.3. An important outcome measure of treatment is whether patients are disease free. Definitions vary, but in our study, we considered patients who had stable EDSS (increase <1.0), no relapses, and no Gd-enhancing or new T2-hyperintense lesions on MRI at 1 year. According to our definition, 63% of the patients were disease free at 1 year. This is similar to the 68% reported in the study by Prosperini et al. [11] using a similar definition.

**Table 4:** Comparison of patients enrolled in observational studies and the AFFIRM trial

Reference	Country/no. centers	No. patients	Duration of treatment	Mean ARR in year prior to treatment	Mean baseline EDSS
Putzki et al. [8]	Germany and Switzerland/5	97 (six previously untreated)	≥12 months	2.3	3.4
Oturia et al. [9]	Denmark/2	234 (175 after switching from DMT, 45 switching from mitoxantrone, 14 treatment naïve)	Median 11.3 months (range 3–21.5 months)	2.53	4.0^a^
Sangalli et al. [10]	Italy/3	285 (233 after switching treatment, 52 treatment naïve)	Up to 2 years	2.13	Not reported
Outteryck et al. [12]	France/not stated^b^	384 (5.6% treatment naïve; efficacy data for 127)	≥12 months	2.19	3.53
Prosperini et al. [11]	Italy/1	190 (efficacy data for 169)	Median 15 (range 1–29 months)	2.0	3.4^a^
Mancardi et al. [15]	Italy/164	2,971	–	–	–
Putzki et al. [16]	Switzerland/3	85 (after failure of DMT)	Median 17.2 (range 12–31.4 months)	2.0	3.1
Piehl et al. [17]	Sweden/36	1,115	Mean 22 months	–	3.86^c^
Fernández et al. [18]	Spain/1	77	Mean 14.7 months	0.96	3.18
Horga et al. [19]	Spain	112	Mean 15.8 months	2.25	4.0
Fernández et al.^d^	Spain/86	1,415	Median 16 (range 1–50)	2.23	3.23
AFFIRM/Polman et al. [4]	Multinational/99	627 (natalizumab)	2 years	1.53 (natalizumab)	2.53
		315 (placebo)		1.50 (placebo)	

*ARR* annualized relapse rate, *DMT* disease-modifying therapy, *EDSS* Expanded Disability Status Scale

^a^Median

^b^Data reported for two regions

^c^For the 363 patients who completed 24 months of treatment

^d^This study

**Table 5:** Comparison of efficacy in observational studies and the AFFIRM trial

Reference	ARR at 1 year	Decrease in ARR at 1 year with respect to previous year (%)^e^	Patients relapse free after 1 year (%)	Change in mean EDSS after 1 year	Patients with EDSS progression (≥1.p point increase) at 1 year (%)
Putzki et al. [8]	0.2	91	80.4	0.4	10
Oturia et al. [9]	0.68	73	63	–	9
Sangalli et al. [10]	0.26	88	84 (78% at 2 years)	–	–
Outteryck et al. [12]	0.59	73	60	0.5	–
Prosperini et al. [11]	0.22	90^a^	85^a^	0.2^c^	–
Putzki et al. [16]	0.27	87	78^b^	0.2	7
Piehl et al. [17]	–	–	–	0.48^d^	–
Fernández et al. [18]	0.13	86	–	0.05	–
Horga et al. [19]	0.24	89	80.3	0.2	9.2
Fernández et al.^f^	0.25	88	80	0.5^e^	6
AFFIRM/Polman et al. [4]	0.26 (natalizumab)	83	77 (natalizumab)	–	13 (natalizumab)
	0.81 (placebo)	54	56 (placebo)		21 (placebo)

*ARR* annualized relapse rate, *EDSS* Expanded Disability Status Scale

^a^Calculated for entire follow-up period (up to 29 months)

^b^Calculated for entire follow-up period (median 17.2 months)

^c^At 15 months follow-up

^d^For the 363 patients who completed 24 months of treatment

^e^Change in median

^f^This study

Our data are also in agreement with the findings to date of the Tysabri Observational Program (TOP), which has enrolled more than 3,000 patients treated with natalizumab in a clinical practice setting [18]. As of June 2010, 2,150 patients had been enrolled (58% with EDSS ≥3.5 compared with 46% in our study). The mean ARR before starting natalizumab was 1.98. After natalizumab treatment, the ARR had decreased to 0.26. A similar picture also emerges from the STRATA study, which enrolled patients who had participated in clinical trials for a further 48 weeks of treatment [19]. Thus the ARR remained low (0.18), even after median total number of natalizumab infusions of 37.

The discontinuation rate in our study was 14%, which is slightly higher than most of the other observational studies (Table 6). This might be partly explained by the longer duration of follow-up than in most other studies. There was one discontinuation due to PML and one due to probable PML. Given that the case of suspected PML was later confirmed, the incidence of PML in our study was 0.15%. This is somewhat higher than in an Italian observational study of the safety data which reported a rate of 0.03% [13] but lower than the 0.27% reported by a surveillance study in Sweden [15].

**Table 6:** Comparison of safety outcomes and the AFFIRM trial

Reference	Discontinuations	Hypersensitivity reactions	Neutralizing antibodies
Putzki et al. [8]	8 (8.2%)	2 (2.1%)^a^	4 (4.1%)^b^
Oturia et al. [9]	27 (12%)	9 (3.8%)	7 (2.9%)
Sangalli et al. [10]	34 (12%)	18 (6.3%)	19 (6.6%)
Outteryck et al. [12]	35 (9.1%)	15 (3.9%)	5 (1.3%)^b^
Prosperini et al. [11]	31 (16.3%)	4 (2.1%)^a^	19 (10%)^c^
Putzki et al. [16]	10 (12%)	2 (2.4%)^a^	6 (7.1%)^b^
Piehl et al. [17]	116 (10.4%)	–	39 (3.9%)
Fernández et al. [18]	13 (16.8%)	1 (1.3%)	9 (11.7%)
Horga et al. [19]	16 (14.4%)	5 (4.5%)	–
Fernández et al.^d^	176 (14%)	46 (3.6%)	34 (3.1%)^c^
AFFIRM/Polman et al. [4]	3.8% (natalizumab)	9% (natalizumab)	57 (9%)
	4.8% (placebo)	4% (placebo)	–

^a^Only reported if leading to discontinuation

^b^Persistent and leading to discontinuation

^c^Only patients with suspected antibodies were tested for neutralizing antibodies. Nevertheless the percentage is calculated with respect to the total population on the assumption that antibodies were only tested in the event of clinical suspicion

^d^This study

In a review of data from clinical trials, Yousry et al. [20] concluded that the risk of PML was approximately 0.1%. This figure, however, included two cases from the SENTINEL clinical trial in which natalizumab was given in combination with interferon beta 1a, and the approved label forbids concomitant treatment with other disease-modifying therapies. As of March 2011, 102 cases of PML had been confirmed, in more than 78,800 patients exposed, corresponding to incidence of approximately 0.1% [21]. According to several studies [22, 23], the risk of PML increases with prior immunosuppressant exposure and treatment duration.

Both cases of PML reported in our study occurred after approximately 1 year of treatment, and both patients had high EDSS scores. One patient had undergone hematopoietic stem cell transplantation. Given its high toxicity, such an intervention has only been considered in patients with refractory disease [24]. Although PML has occasionally been reported as a complication of hematopoietic stem cell transplantation, it is generally considered rare in this type of intervention [25]. Therefore, it is possible that prior hematopoietic stem cell transplantation increases the risk of PML of natalizumab treatment. In the case of the second patient, no risk factors of note were identified and the patient had not received immunosuppressants. An algorithm for risk stratification has recently been proposed, based on prior immunosuppressant therapy, anti-John Cunningham (JC) virus antibody status, and treatment duration >2 years [21]. The antibody status in these patients was unknown. Almost 10% of our patients had received prior immunosuppressive therapy and 21% had received more than 2 years of treatment and none of these had developed PML at the time of data collection, apart from the above-mentioned cases.

In conclusion, the present observational study in a large population provides further support for the efficacy of natalizumab in a clinical practice setting. The sharp decrease in relapse rate in the year following initiation of natalizumab treatment in patients with more severe disease than those included in the pivotal trial was in line with other observational studies performed in different countries, with different methodologies and with different treatment protocols.

## Appendix

### GEXNE Group: authors (hospital) (number of patients included in the study)

Montalbán Gairín, X., Tintoré, M. (H. Universitario Vall d′Hebron - CEM-Cat, Barcelona) (110); Fernández, Ó., Guerrero M., León A, Alonso A., Fernández V., Leyva L. (H. Regional Universitario Carlos Haya, Málaga) (96); Álvarez Lafuente, R., Arroyo González, R., (H. Clínico San Carlos, Madrid) (90); Gamero, M.A., García Moreno, J.M., Izquierdo Ayuso, G., Navarro Mascarell, G., Páramo Camino, M.D., Ruiz-Peña, J.L. (H. Universitario Virgen Macarena, Sevilla) (88); Pérez López, J.L. (H. Sierrallana, Torrelavega) (68); Arbizu Urdiaín, T. (H. de Bellvitge, Barcelona) (55); Málaga, I., Oliva Nacarino, P., Tuñón Álvarez, A. (H. General de Asturias, Oviedo) (41); Ayuso Blanco, T., Bujanda Alegría, M., Lacruz Bescos, F., Soriano Hernández, G. (Complejo Hospitalario de Navarra, H. A, Pamplona) (38); Hernández Clares, R., Meca Lallana, J.E. (H. Virgen de La Arrixaca, Murcia) (35); Hernández, M.A. (H. Universitario Ntra. Sra. de la Candelaria, Tenerife) (32); Aguilar-Amat, M.J., Fernández Pérez, M., Oreja-Guevara, C. (H. Universitario La Paz, Madrid) (31); Ramió i Torrenta, L. (H. Universitario Dr. Josep Trueta, Girona) (28); Agüera Morales, E., Sánchez López, F. (H. Universitario Reina Sofía, Córdoba) (25); Álvarez-Cermeño, J.C., Costa-Frossard, L. (H. Ramón y Cajal, Madrid) (24); Muñoz García, M. (H. Xeral-Cíes, Vigo) (23); Coret Ferrer, F., Navarré-Gimeno A. (H. Clínico Universitario Valencia, Valencia) (22); Asensio Huerga, A.B., Olascoaga, J. (H. Donostia, San Sebastián) (22); de Andrés Frutos, C., Martínez Ginés, M.L. (H. General Universitario Gregorio Marañón, Madrid) (22); Arnal García, C. (H. Virgen de las Nieves, Granada) (22); Millán-Pascual, J., Turpín Fenoll, L. (Complejo Hospitalario La Mancha - Centro, Alcazar de San Juan) (20); Alarcia, R., Ara, J.R., Sánchez-Carteyron, A. (H. Universitario Miguel Servet, Zaragoza) (20); Blanco, Y. Saiz, A. (H. Clinic. IDIBAPS, Barcelona) (19); Pérez Sempere, A. (H. General Alicante, Alicante) (18); Padilla Parrado, F. (H. Clínico Universitario Virgen de La Victoria, Málaga) (17); Argente, J. H. Universitario Puerta Del Mar, Cádiz) (16); Arias, M., Dapena, M.D. (H. Clínico Universitario Santiago de Compostela, Santiago de Compostela) (15); Pérez Ruiz, D. (H. Comarcal El Bierzo, León) (15); Ferias, I., Gracia Gil, J., Rallo Gutiérrez, B. (H. General de Albacete, Albacete) (15); Carrera, D., Ramos, C. (H. Germans Trias i Pujol, Badalona) (15); Barrero, F.J., Fernández-Ortega, J.D. (H. San Cecilio, Granada) (15); Yusta Izquierdo, A. (H. Universitario de Guadalajara, Guadalajara) (15); Oterino Durán, A. (H. Universitario Marqués de Valdecilla, Santander) (15); Suárez Moro, R. (H. Valle del Nalón, Langreo) (14); Fernández-Bolaños Porras, R. (H. Universitario de Valme, Sevilla) (13); Otano, M. (Complejo Hospitalario de Navarra, H. B, Pamplona) (13); Cervelló, A., Parra Martínez, J. (Consorcio H. General Universitario Valencia, Valencia) (12); Fraile, A., Téllez, N. (H. Clínico Universitario de Valladolid, Valladolid) (12); Bonaventura, I. (H. Mutua de Terrassa, Terrassa) (12); Bowakim Dib, W., Tola-Arribas, M.A. (H. Universitario Río Hortega, Valladolid) (12); Munteis, E. (H. del Mar, Barcelona) (11); Íñiguez, C. (H. Clínico Universitario Lozano Blesa, Zaragoza) (10); Arés-Luque, A., Villafani-Echazú, J. (H. de León, León) (10); Iglesias, F. (H. General Yagüe, Burgos) (10); Marzo, M.E. (H. San Pedro, Logroño) (9); Escartin, A., López González, M. (H. Santa Creu i Sant Pau, Barcelona) (9); Marco Igual, M. (H. de Sabadell, Sabadell) (8); Batlle i Nadal, J. (H. Santa Tecla, Tarragona) (8); Calles, C., Nuñez V. (H. Son Dureta, Palma de Mallorca) (8); Mallada Frechín, J. (H. Virgen de la Salud de Elda, Elda) (8); Eguia, P. (H. Doctor José Molina Orosa, Lanzarote) (7); Meca-Lallana, V. (H. Universitario de La Princesa, Madrid) (7); Reyes Yánez, M.P. (H. Universitario Insular de Gran Canarias, Las Palmas de Gran Canaria) (7); Ayuso Peralta, L., Rubio Pérez, L. (H. Universitario Príncipe de Asturias, Alcalá de Henares) (7); Casado, J.L., González Oria, C., Uclés Sánchez, A. (H. Universitario Virgen del Rocío, Sevilla) (7); Caminero, A. (Complejo Hospitalario Ntra. Sra. de Sonsoles, Ávila) (6); Cano, A. (H. de Mataró, Mataró) (6); Berenguer, L., Pérez Carmona, N. (H. Marina Baixa, Villajoyosa) (6); Pato Pato, A. (H. POVISA, Vigo) (6); Guijarro-Castro, C. (H. Universitario 12 De Octubre, Madrid) (6); Miralles Martínez, A. (H. Universitario Infanta Sofía, San Sebastián de los Reyes) (6); Rodríguez García, E. (H. Universitario Severo Ochoa, Leganés) (6); Losada López, M. (Fundación Jiménez Díaz, Madrid) (5); Aguirre Sánchez, J.J. (H. Infanta Cristina, Badajoz) (5); Suárez Fernández, G. (H. Ntra. Sra. del Prado, Talavera de la Reina) (5); Carod-Artal, F.J., López Martínez, A. (H. Virgen de La Luz, Cuenca) (5); García Montero, M.R. (H. Virgen de La Salud, Toledo) (5); Álvarez de Arcaya, A. (H. de Txagorritxu, Vitoria) (4); Gómez Gutiérrez, M. (H. San Pedro de Alcántara, Cáceres) (4); Domínguez-Morán, J.A., Peiró Vilaplana, C. (H. Universitario De La Ribera, Alzira) (4); García-Moncó, C., Mediavillam J., Sánchez Menoyo, J.L. (H. de Galdakao-Usansolo, Galdakao) (3); Landete Pascual, L. (H. Dr. Peset, Valencia) (3); Lacruz Ballester, L. (H. Francesc de Borja, Gandía) (3); Ballabriga Planas, J. (H. Son Llátzer, Palma de Mallorca) (3); Aladro, Y. (H. Universitario de Getafe, Getafe) (3); Castillo, L., Borrega, L. (H. Universitario Fundación Alcorcón, Alcorcón) (3); Perla Muedra, C., Valero Merino, C. (H. Arnau de Villanova, Valencia) (2); Quílez Martínez, A. (H. Arnau de Villanova, Lleida) (2); Sistiaga, C. (H. Bidasoa y Policlínica Gipuzkoa, Hondarribia) (2); Muñoz Málaga, A. (H. de Jerez, Jerez de la Frontera) (2); Navarré-Gimeno, A. (H. de Sagunto, Valencia) (2); González Platas, M. (H. Universitario Canarias, Santa Cruz de Tenerife) (2); Carmona, O. (Fundació Salut Empordá, Figueras) (1); Mendoza Rodríguez, A. (H. General de Segovia, Segovia) (1); Taleti, E. L. (H. General Mateu Orfila, Maó) (1); Romero López, J. (H. Meixoeiro, Vigo) (1); Jarauta Salvador, F. (H. Reina Sofía, Tudela) (1).

## References

[1] Léger OJ, Yednock TA, Tanner L, Horner HC, Hines DK, Keen S (1997). Humanization of a mouse antibody against human alpha-4 integrin: a potential therapeutic for the treatment of multiple sclerosis. Hum Antibodies.

[2] Baron JL, Madri JA, Ruddle NH, Hashim G, Janeway CA (1993). Surface expression of alpha 4 integrin by CD4 T cells is required for their entry into brain parenchyma. J Exp Med.

[3] Stüve O, Bennett JL (2007). Pharmacological properties, toxicology and scientific rationale for the use of natalizumab (Tysabri) in inflammatory diseases. CNS Drug Rev.

[4] Miller DH, Khan OA, Sheremata WA, Blumhardt LD, Rice GP, Libonati MA (2003). A controlled trial of natalizumab for relapsing multiple sclerosis. N Engl J Med.

[5] O’Connor P, Miller D, Riester K, Yang M, Panzara M, Dalton C (2005). Relapse rates and enhancing lesions in a phase II trial of natalizumab in multiple sclerosis. Mult Scler.

[6] Polman CH, O’Connor PW, Havrdova E, Hutchinson M, Kappos L, Miller DH (2006). A randomized, placebo-controlled trial of natalizumab for relapsing multiple sclerosis. N Engl J Med.

[7] Rudick RA, Stuart WH, Calabresi PA, Confavreux C, Galetta SL, Radue EW (2006). Natalizumab plus interferon beta-1a for relapsing multiple sclerosis. N Eng J Med.

[8] Putzki N, Yaldizli O, Mäurer M, Cursiefen S, Kuckert S, Klawe C (2010). Efficacy of natalizumab in second line therapy of relapsing-remitting multiple sclerosis: results from a multi-center study in German speaking countries. Eur J Neurol.

[9] Oturai AB, Koch-Henriksen N, Petersen T, Jensen PE, Sellebjerg F, Sorensen PS (2009). Efficacy of natalizumab in multiple sclerosis patients with high disease activity: a Danish nationwide study. Eur J Neurol.

[10] Sangalli F, Moiola L, Bucello S, Annovazzi P, Rizzo A, Radaelli M (2010). Efficacy and tolerability of natalizumab in relapsing-remitting multiple sclerosis patients: a post-marketing observational study. Neurol Sci.

[11] Prosperini L, Borriello G, Fubelli F, Marinelli F, Pozzilli C (2010). Natalizumab treatment in multiple sclerosis: the experience of S. Andrea MS centre in Rome. Neurol Sci.

[12] Outteryck O, Ongagna J, Zéphir H, Fleury MC, Lacour A, Blanc F (2010). Demographic and clinic characteristics of French patients treated with natalizumab in clinical practice. J Neurol.

[13] Mancardi GL, Tedeschi G, Amato MP, D’Alessandro R, Drago F, Milanese C (2010). Three years of experience: the Italian registry and safety data update. Neurol Sci.

[14] Putzki N, Yaldizli O, Bühler R, Schwegler G, Curtius D, Tettenborn B (2010). Natalizumab reduces clinical and MRI activity in multiple sclerosis patients with high disease activity: results from a multicenter study in Switzerland. Eur Neurol.

[15] Piehl F, Holmén C, Hillert J (2010). Swedish natalizumab (Tysabri) multiple sclerosis surveillance study. Neurol Sci.

[16] Fernández O, Alvarenga MP, Guerrero M, León A, Alonso A, López-Madrona JC (2011). The efficacy of natalizumab in patients with multiple sclerosis according to level of disability: results of an observational study. Mult Scler.

[17] Horga A, Castillo J, Rio J, Tintore M, Auger C, Sastre-Garriga J (2011). An observational study of the effectiveness and safety of natalizumab in the treatment of multiple sclerosis. Rev Neurol.

[18] Belachew S, Butzkueven H, Kappos L, Pellegrini F, Trojano M, Wiendl H et al (2010) Natalizumab (Tysabri^®^) observational program: associations of baseline disease activity and treatment history with postbaseline relapses in multiple sclerosis patients treated with natalizumab. Poster P494, 26th congress of the European committee for treatment and research in multiple sclerosis, Gothenburg, Sweden, 13–16 October 2010

[19] O’Connor PW, Goodman AD, Kappos L, Lublin FD, Polman CH, Rudick RA et al (2010) Updated efficacy and safety of natalizumab in patients who participated in the STRATA study. Poster P483, 26th congress of the European committee for treatment and research in multiple sclerosis, Gothenburg, Sweden, 13–16 October 2010

[20] Yousry TA, Major EO, Ryschkewitsch C, Fahle G, Fischer S, Hou J (2006). Evaluation of patients treated with natalizumab for progressive multifocal leukoencephalopathy. N Engl J Med.

[21] Sandrock A, Hotermans C, Richman S, Natarajan A, Lee S, Plavina T et al (2011) Risk stratification for progressive multifocal leukoencephalopathy (PML) in MS patients: role of prior immunosuppressant use, natalizumab-treatment duration, and anti-JCV antibody status. 63rd annual meeting of the American Academy of Neurology, Honolulu, Hawaii, 9–16 April 2011

[22] Bozic C, Hyde R, Natarajan A, Kooijmans-Coutinho M (2010) Utilization and safety of natalizumab in patients with relapsing multiple sclerosis. Poster P893, 26th congress of the European committee for treatment and research in multiple sclerosis, Gothenburg, Sweden, 13–16 October 2010

[23] Clifford DB, De Luca A, DeLuca A, Arendt G, Giovannoni G, Nath A (2010). Natalizumab-associated progressive multifocal leukoencephalopathy in patients with multiple sclerosis: lessons from 28 cases. Lancet Neurol.

[24] Pasquini MC, Griffith LM, Arnold DL, Atkins HL, Bowen JD, Chen JT (2010). Hematopoietic stem cell transplantation for multiple sclerosis: collaboration of the CIBMTR and EBMT to facilitate international clinical studies. Biol Blood Marrow Transplant.

[25] Coppo P, Laporte JP, Aoudjhane M, Lebon P, Isnard F, Lesage S (1999). Progressive multifocal leucoencephalopathy with peripheral demyelinating neuropathy after autologous bone marrow transplantation for acute myeloblastic leukemia (FAB5). Bone Marrow Transplant.

